# Independent Evaluation of a Commercial AI Software for Incidental Findings of Pulmonary Embolism (IPE) on a Large Hospital Retrospective Dataset

**DOI:** 10.1155/rrp/9091895

**Published:** 2025-03-11

**Authors:** S. Ambrogio, I. Verdon, B. Laureano, K. V. Ramnarine, F. Fedele, D. Vilic, I. Honey, E. Barton, C. Goncalves, Sze Mun Mak, H. Shuaib, A. Jacques

**Affiliations:** ^1^Department of Medical Physics and Clinical Engineering, Guy's and St Thomas' NHS Foundation Trust, London, UK; ^2^Department of Radiology, Guy's and St Thomas' NHS Foundation Trust, London, UK

## Abstract

**Background:** Early treatment of pulmonary embolism is associated with better outcomes, yet incidental PE (IPE) is frequently missed. This retrospective study aims to provide an independent assessment an artificial intelligence (AI) software, developed for flagging IPEs on CT scans.

**Methods:** The study included consecutive CT examinations of 5042 unique patients (8 scanners and 3 protocols) acquired at a large NHS Trust between 01 January 2022 and 30 September 2022. Two radiologists blindly and independently reviewed the AI “positive” and a random selection of “negative” cases to establish the reference standard (*n* = 200). Discrepancies were adjudicated by a third radiologist. The clinical reports of the 200 cases were reviewed for comparison. Performance metrics for the software were calculated for the full (*n* = 5042) and reviewed (*n* = 200) cohorts separately.

**Results:** Based on the reference standard, the IPE prevalence was 1.6% (81/5041). Across the reviewed cohort, the algorithm detected PE with a sensitivity of 96.4%, a specificity of 89.7%, a PPV of 87.1%, an NPV of 97.2%, and an accuracy of 92.5%. Across the full cohort, the algorithm exhibited a sensitivity of 96.4%, a specificity of 99.8%, a PPV of 87.1%, an NPV of 99.9%, and an accuracy of 99.7%. A review of the original clinical reports indicated that 11 cases of IPE were initially unreported. A total of 34 examinations were rejected by the software. While the scanner performed consistently across patient sexes and ethnicities, discrepancies were found among CT scanners.

**Conclusions:** The AI software detected IPE with a high diagnostic accuracy on a large NHS dataset, showing that AI–supported reporting could improve diagnostic accuracy and reduce times to diagnosis.

## 1. Introduction

A high record of approximately 7.5 million patients are currently on the waiting list for hospital treatment while the NHS is facing the biggest workforce crisis in its history [1]. Worldwide, the WHO has predicted a shortage of 14 million healthcare workers by 2030 [2], meanwhile the demand for healthcare services is constantly growing, widening the gap between demand and supply. Data suggest an ongoing shortage of radiologists amid a steep increase in workload: CT scanning activity increases each year in the NHS: in 2024, this was by 8.4% [3], and in 2024, a 30% shortfall in NHS clinical radiologists was reported with workforce number being predicted to decrease further [4]. This is likely to contribute to delayed diagnosis, radiologist burnout, and increasing errors due to fatigue and biases [3, 5].

Digital technologies play a key role in today's economy and have the potential to radically improve the quality of care, reducing costs and inequalities [6]. The use of artificial intelligence (AI) in radiology has the potential to support clinicians by increasing diagnostic accuracy and improving efficiency in clinical workflows while reducing the radiologist's workload [7]. AI algorithms have been demonstrated to be valuable as triage tools in radiology to flag certain pathologies and prioritize urgent cases in worklists, accelerating time to treatment and decreasing the risk of adverse events [7–11].

Pulmonary embolism (PE) arises due to blood clots in the pulmonary arteries and is the third most common cause of cardiovascular death worldwide [12]. Risk factors for PE include patient immobility, pregnancy, underlying malignancy, obesity, hypertension, and diabetes in addition to hemostasis disorders [12, 13]. Prompt diagnosis and treatment with anticoagulation therapy are associated with better outcomes [14]. Accurate diagnosis is also vital to avoid unnecessary anticoagulation and potential associated bleeding risks. When clinically suspected, the diagnosis is most commonly confirmed with CT pulmonary angiogram (CTPA) using a protocol optimized for opacification of the pulmonary arterial tree and visualization of PE. Several AI algorithms have been developed to detect and triage PEs on CTPAs [15, 16] with high sensitivity and specificity [17, 18] and have been shown to reduce waiting times [11].

However, the disease can also be detected incidentally on contrast-enhanced CT scans of the thorax performed for other reasons; such instances are referred to as incidental PE (IPE) [19]. A meta-analysis of 12 studies, including a total of more than 10,000 patients, estimated that IPE occurs with a reported frequency of 2.6% [20]. However small, distal PEs are often missed by radiologists. Additional retrospective review studies suggested that between 50% and 70% of IPEs were originally unreported in clinical settings [21, 22]. Consequently, the implementation of AI models to improve IPE detection in routine chest CT examinations has been investigated across several studies [23–27]. However, due to the low prevalence of the disease and poor visualization of PE in standard chest CT protocols, further studies on larger datasets are required to assess the clinical relevance of such AI algorithms to the general population [24].

A commercially available, FDA–approved, and CE–marked AI algorithm for detecting IPE on routine chest CT examinations (i.e., non-CTPA protocol) has shown promising diagnostic accuracy in two retrospective studies. Batra et al. [24] demonstrated that the model's sensitivity was comparable to the original clinical reports in a dataset of 3003 consecutive conventional chest CT examinations. In another study, Langius-Wiffen et al. [25] found that the AI tool detected 25 cases of IPE that were initially missed by the clinical reports on a sample of 3089 consecutive chest CT examinations, indicating the model to be more sensitive than the clinical report. Beyond improving diagnostic accuracy, the AI tool has shown a potential to decrease the time to diagnosis: a 15-week prospective study on oncology patients reported that the AI tool significantly reduced IPE detection and notification time from 7714 min to 87 min [26]. Moreover, demonstrating the diagnostic accuracy of an AI algorithm is not equivalent to evidencing its clinical efficacy [7]. Indeed, frameworks published to guide the evaluation of diagnostic AI software packages emphasize the need to consider their cost, resource impact, and health benefits relative to standard care, in addition to their usability, compliance with regulations, and validity to ensure safe use and support from service-users and patients [28–30].

The objective of this study is to provide a retrospective, independent assessment of the CE–marked and FDA–approved AI algorithm for IPE detection (Aidoc, Tel Aviv, Israel) on clinical data acquired at a large NHS Trust (Guy's and St Thomas' NHS Foundation Trust) to determine the model's applicability as a decision support tool for radiologists and potential improvement in time to diagnosis and patient safety. Well-documented case studies in real clinical settings are crucial to demonstrating the potential value and challenges of AI tools in improving health outcomes, increasing efficiency, and integrating into clinical workflow. Such examples will help accelerate the effective and safe implementation of artificial intelligence into healthcare systems.

## 2. Methods

### 2.1. Patient Inclusion

CT scans of 5042 unique adult patients (≥ 18 years) were included in the retrospective study. The scans were performed consecutively between 01 January 2022 and 30 September 2022 using 8 different CT scanners from 2 manufacturers (Table 1). The protocols included CT thorax, abdomen, and pelvis with contrast (CCHAPC), CT thorax and abdomen with contrast (CCABDC), and CT thorax with contrast. When available, the ethnicity and sex of the patient cohort were analyzed to address potential bias in the results. Patient ethnicities were recorded following the mandated ethnic monitoring questions and response codes outlined in the NHS data dictionary [31]. Their distribution was consistent with data provided by the Office of National Statistics Census 2011–2021 [32] regarding the London boroughs served by Guy's and St Thomas' NHS Foundation Trust (Southwark, Lambeth, and Lewisham). The total number of images (*n*) and the percentage (percentage, %) with respect to the total dataset are reported in Table 1.

### 2.2. Data Management

The identified CT scans were extracted from the radiology PACS system (Sectra AB) and imported into XNAT (https://www.xnat.org), an open-source imaging-based research platform. The data were anonymized in accordance with DICOM Supplement 142 (https://www.dicomstandard.org). The data extraction was automated and manually spot-checked for data quality assurance. XNAT at Guy's and St Thomas' NHS Foundation Trust is held within a secure enclave behind the firewall to ensure data safety and access was only granted to the scientific lead of the retrospective study.

The anonymized data remained linked to the original data in a lookup table stored separately by the study lead to ensure that AI results could be compared to the original report. Once extracted and checked for quality, the data were sent directly from XNAT to the AI application for processing. No data were excluded. Upon the completion of the study, the data were deleted from XNAT.

### 2.3. AI Algorithm

All anonymized CT scans were analyzed by the CE–marked and FDA–approved incidental PE algorithm developed by Aidoc (Aidoc, Tel Aviv, Israel). The basis of the AI algorithm is described in detail by a similar study by Langius-Wiffen et al. [25]. The algorithm was deployed on a dedicated virtual machine with a direct connection to the XNAT DICOM node. The images were automatically forwarded in batches and analyzed by the software in the cloud. Cloud processing relies on providers which are ISO 27001 certified, ensuring data security and compliance with GDPRs. The system complies with the System Level Security Policy (SLSP) of Guy's and St Thomas' NHS Foundation Trust in accordance with NHS information security policy and standards. After processing all the images, the algorithm returned a list of the results in a binary classification form: “positive” and “negative.” The algorithm was not trained or fine-tuned on the retrospective dataset.

### 2.4. Reference Standard

The reference standard on the CT scans was established by reviewing the cases classified by the algorithm as “positive” (approximately 2% or 93 cases) and a random selection of 107 cases classified as “negative.” A total of 200 cases were reviewed, blindly, by two consultant radiologists working at Guy's and St Thomas' NHS Foundation Trust with extensive experience in the specific field. A third consultant radiologist working in the Trust blindly and independently adjudicated discrepancies to confirm the presence or absence of PE. Given the workforce shortages and unavailability of funding, the choice of 200 cases was agreed to compromise between achieving meaningful statistics and affecting the work–life balance of the radiologists. A data processing agreement (DPA) was signed by both Guy's and St Thomas' NHS Foundation Trust and Aidoc to ensure the processing and management of data in compliance with the instruction of the data controller.

### 2.5. Statistical Analysis

The sensitivity, specificity, positive predictive value (PPV), negative predictive value (NPV), and accuracy of both the AI software and the initial clinical reports were calculated for the reviewed and entire cohorts (200 and 5042 cases, respectively). The latter was performed by assuming that unreviewed negatives were true negatives (TNs). 95% Confidence intervals (CIs) were obtained using the “exact” Clopper–Pearson CI method [33] in *R* (Version 4.4.0). Sensitivities and specificities were stratified by patient sex, scanner protocol, and scanner model using Fisher's exact tests in *R* (Version 4.4.0). Statistical significance was set to *p* > 0.05. The sample size (5042 cases) was calculated to be sufficient based on performance metrics and an IPE incidence from a similar study (95% CI and 5% precision) [25, 34].

## 3. Results

### 3.1. Data Processed

**Figure 1:**
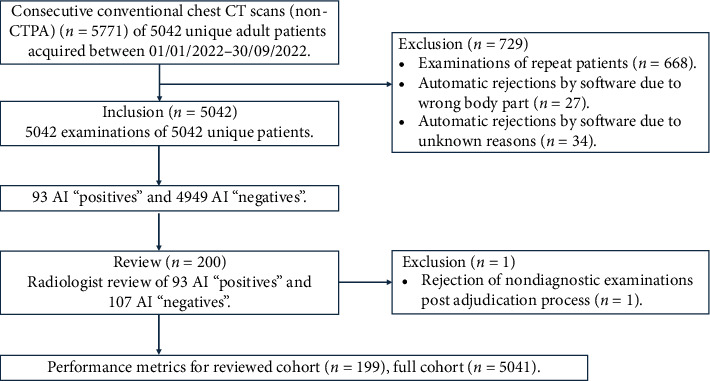
Flowchart of study inclusions and exclusions. AI = artificial intelligence; CT = computed tomography; CTPA = CT pulmonary angiogram.

The software analyzed a total of 5042 consecutive CT examinations of unique patients (age > 18 years old). The algorithm returned a total of 93 “positive” cases, or an IPE incidence of 1.8% in the patient cohort. The study workflow is summarized in Figure 1. About 1.2% of scans (*n* = 61) were automatically rejected or not analyzed by the software. By reviewing the rejected examinations, it was determined that 27 cases (44.6%) were rejected because they contained the wrong body part, while 34 cases (55.7%) were rejected for unknown reasons. Consequently, the software's actual rejection rate was established to be 0.67% (*n* = 34). The distribution of rejections across scanners and protocols reflects the original cohort distribution reported in Table 1.

### 3.2. Diagnostic Accuracy Algorithm

A total of 200 images, consisting of the 93 “positive” cases in addition to 107 randomly selected “negative” cases, were blindly reviewed by the radiologists. One “negative” case was excluded from the cohort, as the clinicians indicated that the CT acquisition included just the base of the lungs and was not suitable for diagnosing a PE. Following the review, the algorithm exhibited a sensitivity (true positive rate or recall) of 96.4% (95% CI: 89.9%–99.3%), a specificity (TN rate) of 89.7% (95% CI: 82.6%–94.5%), a PPV (precision) of 87.1% (95% CI: 79.8%–92.0%), a NPV of 97.2% (95% CI: 91.9%–99.1%), and an overall accuracy of 92.5% (95% CI: 87.9%–95.7%). A batch analysis method was used to reduce the review burden. However, this induced highly biased sampling, which may reflect a worst-case scenario and significantly impact the specificity, NPV, and overall accuracy of the results. Considering the whole cohort, the algorithm exhibited a sensitivity of 96.4% (95% CI: 89.9%–99.3%), a specificity of 99.8% (95% CI: 99.6%–99.9%), a PPV of 87.1% (95% CI: 79.8%–92.0%), a NPV of 99.9% (95% CI: 99.8%–100.0%), and an overall accuracy of 99.7% (95% CI: 99.5%–99.8%). By reviewing the occurrence of false positives (FPs) and false negatives (FNs), 5 out of 12 FPs were flagged as “equivocal” or with “poor contrast” by the radiologists. Furthermore, discrepancies between the clinicians were identified for threetwo of these cases, one corresponding to the image quality being suboptimal for identifying a PE, and another a complex imaging case. For the 3 FNs, comments from the radiologist review identified factors that could obscure or mimic PE findings, including one case with a right chronic web and another with a tumor thrombus invading the pulmonary artery.

Contingency tables for both the reviewed and the entire cohorts are presented in Table 2 In addition, performance tables for the reviewed and whole cohorts are shown in Table 3.

### 3.3. Technology

The software's performance showed no statistically significant association with the protocol (*p* > 0.05). Although there was no statistically significant difference in the sensitivities (*p* > 0.05) of the scanner models, there was a statistically significant difference in their specificities (*p*=0.02). A pairwise exact Fisher's test was used to establish that the Siemens Healthineers SOMATOM Force CT scanner had a significantly higher specificity than the Siemens Healthineers SOMATOM Edge scanner model (99.9% vs. 99.4%, *p*=0.01), and that the specificity of the Philips Brilliance iCT scanner was not significantly different to that of the 2 Siemens scanners. The sensitivity and specificity of the AI software, stratified by subgroups, are outlined in Table 4 with the outcomes of the Fisher's exact tests.

Peak kilovoltage (kVp) used in the examinations ranged between 90 kVp and 120 kVp across scanner models and protocols. Despite an apparent weak relationship between lower dose exams and “positive” findings, it was not possible to confirm this with any statistical significance.

### 3.4. Patient Demographic

The AI software's performance did not vary significantly in association with patient sex (*p* > 0.05), as shown in Table 4. The distribution of “positive” cases across several ethnic groups was not suitable for meaningful statistical tests but appears consistent with the distribution of ethnicities across the whole cohort, as outlined in Table 1, where the number of “positive” cases in an ethnic group was less than 5 and *n* was denoted as *n* < 5 or 5% to ensure patient anonymity.

### 3.5. Comparison With the Original Reports

The original clinical reports from the examinations were anonymized and then reviewed by the radiologists. A total of 11 “positive” PE findings were missed in the original reports and confirmed positives during the secondary review. In addition, the time between the CT scan and the clinical report was recorded to estimate the time to diagnosis. The Guy's and St Thomas' NHS Foundation Trust has an average turnaround time for reporting CT scans of 4.5 days with peaks of over 20 days. The AI tool can significantly reduce the time to diagnosis to the same day (within 24 h) with potential improvement to patient safety and outcomes.

## 4. Discussion

IPEs can be life-threatening and occur more commonly in oncology patients [35]. Delayed diagnosis and treatment of IPEs can lead to worsening clot burden, associated morbidity, poorer patient outcomes, and reduced overall survival [36, 37]. Conversely, early identification and treatment (i.e., therapeutic anticoagulation) are associated with reduced mortality, even in acute cases [14, 38]. Guy's and St Thomas' NHS Foundation Trust has a large oncology patient cohort and in 2022, had an average CT reporting time of 4.5 days with peaks of more than 20 days during periods of reduced radiologist capacity. This potentially could have had a negative impact on patient outcomes. Reporting backlogs are expected to rise with increasing imaging demand compounding radiologist shortages. Furthermore, periods of industrial action are likely to exacerbate the issue [39]. The deployment of promising AI–Imaging solutions to accelerate diagnosis and improve patient outcomes is United Kingdom's National priority, as demonstrated by recent funding streams for the adoption of such technologies [40]. A growing number of algorithms with applications for early detection or triage, incidental findings, diagnosis, treatment management, and monitoring are being developed [9]. However, just a few algorithms have been successfully deployed into clinical practice. Practical challenges remain, and pilot studies are crucial to support healthcare organizations in adopting AI technologies by providing essential knowledge [41, 42].

This study investigated the performance of a commercial, FDAI–approved, and CEI–marked AI software (Aidoc, Tel Aviv, Israel) for detecting IPEs on 5042 consecutive nondedicated chest CT examinations retrospectively collected at Guy's and St Thomas' NHS Foundation Trust. A previous study has already demonstrated the potential of the algorithm to significantly reduce waiting times by prioritizing radiologist worklists [26]. Instead, this retrospective evaluation is part of a potential procurement exercise, as recommended by the guidelines published by NHSX [28].

The algorithm returned 93 “positive” cases. After comparing with the reference standard, 12 cases were FPs and the final IPE prevalence was confirmed to be 1.6%. Previous studies have reported similar values, with IPE prevalence ranging from 1.3% to 2.6% [20, 24, 25, 38]. The AI algorithm identified IPE with a high sensitivity of 96.4% (95% CI: 89.9%–99.3%) and a high specificity of 89.7% (95% CI: 82.6%–94.5%). The AI tool produced 12 FPs, giving a PPV of 87.1% (95% CI: 79.8%–92.0%) and 3 FNs, leading to a high NPV of 97.2% (95% CI: 91.9%–99.1%). The rigorous review process was independently performed by 2 consultant radiologists in a blind study. Discordance between readers was adjudicated by a third radiologist to determine the ground truth. Similarly to other studies, the review process was performed by reviewing approximately 2.2% of the “negative” cases [24, 25]. The software's performance was not influenced by patient sex, and the distribution of ethnicities across the “positive” cases was comparable to those of the original dataset. This indicates that there are likely no significant biases embedded in the algorithm ruling the classification I.

Interestingly, 11 IPE cases flagged by the AI algorithm were missed in the original reports. The 11 cases were confirmed as positive IPEs by both the reference standard and a secondary review by the radiologist, highlighting that the AI solution has the potential to improve the diagnostic accuracy of the department. Considering the discrepancies between the AI output and the radiologist review, poor contrast potentially contributed to 42% of the FPs (5/12), and information from the original clinical reports suggested that the diagnosis was also either consistent or suspicious for PE. However, we did not request an expert radiological assessment nor did we track the patient's clinical histories, as such actions could raise ethical concerns. For the 2/3 FNs, the reviewing radiologists identified pathologies such as right chronic web and thrombi, which may have obscured or mimicked PE findings. These observations highlight the challenges and limitations of AI–driven technology in such complex cases and underscore the importance of a multidisciplinary approach that combines AI, expert radiological interpretation, and clinical context to enhance diagnostic accuracy and improve patient care.

Four additional studies assessed the performance of the same software on cohorts of both nononcology and oncology patients. Batra et al. [24] reported the algorithm to exhibit a higher specificity (99.8%), but lower sensitivity (82.5%) and a comparable PPV (86.8%) and NPV (99.8%) on a dataset of 3003 routine chest CTs. The lower sensitivity might reflect a lower IPE prevalence (1.3%) in their cohort [24, 25]. Similarly, neither sensitivity nor specificity was found to vary significantly with age, sex, or cancer clinical scenario [24]. The study concluded that the software could be valuable for patient triage for early intervention or act as a second reader on CT scans. Wiklund, Medson, and Elf [23] found that the tool performed with a high specificity (99.8%) and sensitivity (90.7%) in a cohort with a higher IPE prevalence (4.0%). In their study, the algorithm detected 59 IPEs that were initially unreported. However, only data acquired from one scanner were used in this study. Another study, carried out by Langius-Wiffen et al. [25], evaluated the software on a cohort of 3089 routine contrast–enhanced chest CTs where IPE occurred with a frequency of 2.2%. Compared to our findings, they reported the algorithm performed at a slightly lower sensitivity of 95.5%, and a significantly higher specificity of 99.6%, with a comparable PPV of 85.3% and a significantly higher NPV of 99.9%. The tool was able to detect an additional 25 PE cases that were initially missed by radiologists, highlighting the potential of this tool in reducing the rates of missed IPE. Finally, Topff et al. [26] carried out both a retrospective and prospective evaluation of the software where IPE occurred with a prevalence of 1.3% and 1.0% respectively, finding it to perform with greater diagnostic accuracy than the reporting radiologists (91.6% sensitivity and 99.7% specificity). Their study was performed using data from a range of scanners from Toshiba, Siemens, and Philips; however, they did not specify whether the software performed differently across these. Notably, Topff et al. [26] found that IPE detection and notification time decreased from several days (7714 min) with routine workflow to just 87 min with AII–assisted triage. Furthermore, though focusing on PE detection in CTPAs, Rothenburg et al. demonstrated a similar AI triage system to reduce waiting times, despite not significantly improving diagnostic accuracy [11], highlighting the practical benefits of AI triage in radiology.

Our retrospective evaluation adds knowledge to previous literature by reporting findings from a large United Kingdom's hospital using data acquired from different scanner manufacturers and acquisition protocols. First, we report that approximately 0.6% of the images (34 out of 5771) were rejected by the software. This is considerably lower than the 7.3% rejection rate previously reported by Topff et al. [26]. The low number of rejected examinations did not allow for a robust statistical analysis across scanner models, manufacturers, and protocols. However, overall, the distribution of rejected examinations across protocols was consistent with the distribution of the original cohort.

A statistically significant association was observed between the software's performance and scanner models, with the Siemens Healthineers SOMATOM Force CT performing with a significantly higher specificity compared to the Siemens Healthineers Edge model. This difference could be attributed to various factors, including aspects of the CT scanners such as image reconstruction kernels and detector configurations, as well as elements of the AI algorithm, such as the training dataset and mathematical convolution processes. A comprehensive investigation into these factors lies beyond the scope of this paper and would necessitate substantial collaboration with the manufacturers. No statistically significant differences were found between either of the Siemens scanners and the Philips Brilliance iCT scanner, despite this scanner returning 5% of the AI “positive” cases but only contributing to only 9% (*n* = 542) of the total dataset. It was not possible to determine a cause for the significant difference, but the distribution of scans across the protocols and models showed considerable variation, which may have reduced the statistical power of the analyses. Furthermore, it was not possible to establish a significant correlation between tube voltage (kVp) exposure factors and “positive” findings. Potential reasons for the performance discrepancies could include scanner age and reconstruction algorithms. Siemens scanners use the latest generation advanced modeled iterative reconstruction algorithms (ADMIRE), while the Philips Brilliance iCT relies on a first generation iterative reconstruction algorithm (IDOSE). Further studies are required to assess potential biases related to scanner models and exposure parameters to ensure the generalizability of the AI algorithm. It would also be valuable to assess the impact of patient size and image quality on the software's “positive” findings and rejection rates. Parameters such as noise or contrast-to-noise ratio could be useful metrics for assessing these.

This study has some limitations. First, the number of FNs and TNs could be underestimated, as only 2.2% of the “negative” cases were reviewed. Considering the whole cohort, the specificity, NPV, and accuracy could have increased to 99.8% (95% CI: 99.6%–99.9%), 99.9% (95% CI: 99.8%–100.0%), and 99.7% (95% CI: 99.5%–99.8%), respectively. Manual annotation and independent case review are labor-intensive and extremely time-consuming, requiring clinical specialists to meticulously review each examination. Given the shortages in the workforce, batch review is commonly found in other studies in the literature [24–26], as a complete review of 5042 cases would be not feasible. Second, the low prevalence of the pathology (1.6% or 81 cases) could have limited the assessment of biases in the study. These CT scans had a typical size of ∼800 MB and cloud processing of 5042 examinations proved to be resource intensive. Third, it was not possible to investigate the reasons why the 34 images were rejected nor why the different scanners performed differently. This is beyond the scope of this independent evaluation and it would require extensive studies and proprietary information by manufacturers such as Aidoc, Siemens, and Philips. Finally, the author did not perform a comparison between the original reports and the AI findings on the whole dataset. Natural language processing (NLP) and large language models (LLMs) are increasingly used to extract clinical insights from electronic healthcare records; however, validation of such tools is needed as the large presence of free text and unstructured data is still hindering the full utilization of such techniques [24, 43, 44]. Manual review of the cases would be time-consuming and again, require specialist clinical knowledge. This is not feasible in a public hospital without dedicated funding.

Overall, the performance of the software on the retrospective dataset was found satisfactory by the clinical team. Importantly, the AI solution detected 11 IPE cases that were not documented in the clinical reports, highlighting its potential as a valuable tool for improving diagnostic accuracy, speeding time to diagnosis, and enabling prompt intervention with improved patient outcomes. A prospective study will be conducted to clarify the clinical relevance of the software, including where this could be an effective aid in prioritizing and triaging time-sensitive findings and the added benefits of being used in combination with radiologists.

## 5. Conclusion

An independent evaluation of a CE–marked and FDA–approved AI algorithm for IPE detection (Aidoc, Tel Aviv, Israel) was performed on a retrospective cohort of 5042 patients at Guy's and St Thomas' NHS Foundation Trust. Overall, the software performed with high sensitivity, specificity, PPV, and NPV and detected 11 IPE cases not recorded in the original clinical reports. The AI solution could be a valuable tool for improving the diagnostic accuracy of the department, speeding time to diagnosis, and enabling prompt intervention with improved patient outcomes. A prospective evaluation will be conducted to quantify the improvements in time to diagnosis, determine the added benefits of its combined use with radiologists, and compare clinical outcomes with standard care. Postdeployment monitoring in clinical settings will assess the clinical relevance of the software, including its effectiveness in prioritizing and triaging time-sensitive findings.

## Figures and Tables

**Table 1:** Summary of patient and examination characteristics across the full dataset (*n* = 5771) and the 93 AI “positive” cases.

Variable	Value	
	Full dataset	AI “positives”
Scanner model		
Siemens Healthineers SOMATOM Force CT	4095 (71%)	70 (75%)
Siemens Healthineers SOMATOM Edge	1134 (20%)	18 (19%)
Philips Brilliance iCT	542 (9%)	5 (5%)
Protocol		
CT thorax, abdomen, and pelvis with contrast (CCHAPC)	4436 (77%)	76 (82%)
CT thorax and abdomen with contrast (CCABDC)	548 (9%)	5 (5%)
CT thorax with contrast (CCHESC)	787 (14%)	12 (13%)
Sex		
Male	2785 (48%)	48 (52%)
Female	2986 (52%)	45 (48%)
Ethnicity		
White British	2124 (40.6%)	41 (44.1%)
White Irish	134 (1.9%)	< 5 (< 5%)
White-other background	348 (6.1%)	< 5 (< 5%)
Mixed White and Black Caribbean	57 (0.4%)	0 (0%)
Mixed White and Black	50 (0.3%)	< 5 (< 5%)
Mixed White and Asian	52 (0.3%)	< 5 (< 5%)
Mixed-other background	47 (0.3%)	< 5 (< 5%)
Indian-Asian/Asian British	93 (1.1%)	0 (0%)
Pakistani-Asian/Asian British	44 (0.2%)	0 (0%)
Bangladeshi-Asian/Asian British	58 (0.5%)	0 (0%)
Asian-other background	70 (0.7%)	0 (0%)
Caribbean-Black/Black British	263 (4.4%)	5 (5.4%)
African-Black/Black British	238 (4.0%)	6 (6.5%)
Black-other background	127 (1.8%)	< 5 (< 5%)
Chinese	75 (0.8%)	0 (0%)
Any other ethnic group	200 (3.2%)	0 (0%)
Not given-not stated/unknown	1786 (30.9%)	29 (31.2%)

**Table 2:** Contingency table for reviewed cohort (*n* = 199) and full cohort (*n* = 5041).

AI output	Ground truth positive	Ground truth negative
AI “positive”	81 (81)	12 (12)
AI “negative”	3 (3)	103 (4945)

*Note:* Values are presented as reviewed cohort results (full cohort result).

**Table 3:** Clinical performance metrics and results of the AI software for the reviewed and the full cohorts, with 95% CI in parentheses.

Metrics	Reviewed cohort (%)	Full cohort (%)
Sensitivity (true positive rate or recall)	96.4 (89.9–99.3)	96.4 (89.9–99.3)
Specificity (true negative rate)	89.7 (82.6–94.5)	99.8 (99.6–99.9)
Positive predictive value (precision)	87.1 (79.8–92.0)	87.1 (79.8–92.0)
Negative predictive value	97.2 (91.9–99.1)	99.9 (99.8–100.0)
Accuracy	92.5 (87.9–95.7)	99.7 (99.5–99.8)

**Table 4:** Clinical performance metrics stratified by subgroups, 95% CI in parentheses, with outcomes of Fisher's exact tests.

Subgroup	Sensitivity (%)	*p*	Specificity (%)	*p*
Scanner model				
Siemens Healthineers SOMATOM Force CT	97.0 (89.5–99.6)		99.9 (99.7–99.9)	
Siemens Healthineers SOMATOM Edge	91.7 (61.5–99.8)		99.4 (98.7–99.7)	
Philips Brilliance iCT	100.0 (47.8–100.0)		100 0.0 (99.3–100.0)	
		0.50		0.02

Protocol				
CT thorax, abdomen, and pelvis with contrast (CCHAPC)	97.1 (89.9–99.6)		99.8 (99.6–99.9)	
CT thorax and abdomen with contrast (CCABDC)	83.3 (35.9–99.6)		100.0 (99.3–100.0)	
CT thorax with contrast (CCHESC)	100.0 (63.1–100.0)		99.5 (98.6–99.9)	
		0.23		0.12

Sex				
Male	97.5 (86.8–99.9)		99.7 (99.4–99.8)	
Female	95.3 (84.2–99.4)		99.9 (99.6–100.0)	
		1.00		0.17

## Data Availability

The study is an independent evaluation of an AI medical device software conducted in a clinical setting to assess its performance. The dataset consists of nonpublic clinical records from patients at Guy's and St Thomas' NHS Foundation Trust. These data are stored within the hospital's Radiology Information System, Picture Archiving and Communication System (PACS), and Electronic Healthcare Record (HER) systems, with access strictly limited to authorized staff, in compliance with GDPRs. The data are not publicly available due to ethical and privacy reasons.
